# Feasibility of oral HIV self-testing in female sex workers in Gaborone, Botswana

**DOI:** 10.1371/journal.pone.0259508

**Published:** 2021-11-08

**Authors:** Emily Shava, Laura M. Bogart, Kutlo Manyake, Charlotte Mdluli, Kamogelo Maribe, Neo Monnapula, Bornapate Nkomo, Mosepele Mosepele, Sikhulile Moyo, Mompati Mmalane, Till Bärnighausen, Joseph Makhema, Shahin Lockman

**Affiliations:** 1 Botswana Harvard AIDS Institute Partnership, Gaborone, Botswana; 2 Harvard T.H. Chan School of Public Health, Boston, Massachusetts, United States of America; 3 RAND Corporation, Santa Monica, California, United States of America; 4 Nkaikela Youth Group, Gaborone, Botswana; 5 Ministry of Health & Wellness, Gaborone, Botswana; 6 University of Botswana, Gaborone, Botswana; 7 University of Heidelberg, Heidelberg, Germany; 8 Wellcome Trust Africa Centre for Health and Population Studies, Mtubatuba, South Africa; 9 Brigham and Women’s Hospital, Boston, Massachusetts, United States of America; University of California, UNITED STATES

## Abstract

**Background:**

Oral HIV self-testing (HIVST) may be useful for increasing testing in persons at elevated risk of acquiring HIV.

**Methods:**

We conducted a pilot study to evaluate the feasibility (defined by uptake) of HIVST among FSW in Gaborone, Botswana. FSW age 18 years and above were recruited through a non-governmental organization serving FSW. FSW with unknown or negative HIV status at screening performed HIVST in the study clinic following brief training. FSW testing HIV-negative were each given two test kits to take home: one kit to perform unassisted HIVST and another to share with others. Feasibility (use) of HIVST (and sharing of test kits with others) was assessed in these women at a study visit four months later.

**Results:**

Two hundred FSW were screened. Their average age was 34 years (range 18–59), and 115 (58%) were HIV-positive. Eighty-five (42%) tested HIV-negative at entry and were eligible to take part in the HIVST pilot study. All 85 (100%) agreed to take home HIVST kits. Sixty-nine (81%) of these 85 participants had a follow-up visit, 56 (81%) of whom reported performing HIVST at a mean of three and half months after the initial visit. All 56 participants who performed HIVST reported negative HIVST results. Fifty (73%) of the 69 participants who took HIVST kits home shared them with others. Of the 50 women sharing HIVST kits, 25 (50%) shared with their non-client partners, 15 with a family member, 8 with friends, and 3 with a client. One participant did not test herself but shared both her test kits. Most participants 53/56 (95%) found oral HIVST very easy to use whilst 3/56 (5%) felt it was fairly easy.

**Conclusion:**

Oral HIVST is feasible among FSW in Gaborone, Botswana. The majority of FSW used the HIVST kits themselves and also shared extra HIVST kits with other individuals.

## Introduction

HIV infection remains a major public health challenge, particularly in the sub-Saharan region [1, 2]. Heterosexual contact is the main mode of transmission in the region [3], with sex work a significant contributor [4]. Female sex workers are a key population for HIV because of their increased vulnerability to infection [4, 5]. Various HIV prevention strategies need to be employed in order to reach these key populations [6]. Female sex workers (FSW) in sub-Saharan Africa have a very high burden of HIV [4, 7], with HIV prevalence generally at least three times that of the general population [8–12]. HIV testing is a significant barrier to HIV prevention and care in FSW [13–16], and HIV self-testing could potentially provide a means to improve HIV testing coverage among FSW [17–19]. HIV self-testing among these women at increased vulnerability for both HIV and for marginalization may be an important element of HIV prevention and engagement in care [2, 3, 20, 21]. HIV self-testing is generally perceived to be convenient, reducing some of the known barriers to HIV testing among marginalized key populations [22–24]. Stigma and fear of discrimination by healthcare providers represent some of the previously identified barriers [23, 25].

HIV self-testing has not only been shown to be safe but to also increase frequency of testing among some key populations including FSW [19, 26]. In 2012, the US FDA approved Oraquick as the first oral fluid self-administered test for HIV [27]. Following the WHO recommendation for HIV self-testing in 2016, more countries in Sub-Saharan Africa are recommending HIV self-testing in governmental guidelines [4, 28].

Individual HIV self-testing, coupled with secondary distribution of HIV self-test kits, have been shown to be effective HIV prevention strategies in some hard to reach populations in sub-Saharan Africa [29–34]. Studies elsewhere, have shown both oral and blood based HIV self-testing methods to be acceptable in high risk populations [35], with a preference for the former because it is painless and perceived to be easier [36]. Some studies have also concluded that FSW may require some form of training in order for then to correctly perform unassisted HIV self-testing [37]. In Botswana, we previously conducted a qualitative study among FSW in Gaborone to explore the acceptability of HIV self-testing and of secondary distribution of HIV self-test kits [38]. The qualitative study showed that, despite having no prior knowledge of HIV self-testing, female sex workers in Gaborone perceived it to be a highly acceptable method of HIV testing. The research signaled the need for addressing identified structural barriers in the healthcare system in the implementation of HIV self-testing. It further highlighted the importance of having a FSW peer-driven approach when implementing self-testing in this key population, with some oversight and guidance from healthcare professionals [38]. The actual uptake of HIV self-testing has not been evaluated in FSW in Botswana, who are at increased risk for HIV [39].

Against this background we aimed to evaluate the feasibility (uptake) of oral HIV self-testing among FSW (and sharing of HIVST kits with others) in Gaborone, Botswana.

## Methods

### Study design

We conducted a pilot study among FSW in Gaborone Botswana between June 2019 and February 2020 to evaluate the feasibility of oral HIV self-testing. Feasibility was defined as uptake of the intervention (described in detail in “Outcomes” section below) [40, 41]. We used results from prior qualitative work to design the approach to offering self-testing in this study [38].

### Ethical statement

The “Ikitse Study “(“know yourself” in Setswana) was approved by the following institutional review boards: the Health Research and Development Committee of the Botswana Ministry of Health and Wellness (HRDC#00842), and the Office of Human Research Administration at the Harvard T.H. Chan School of Public Health (IRB18-1351). All participants provided written informed consent.

### Participants

To be eligible for the study, participants needed to be 18 years-old or older and identify as a FSW. Specifically, women needed to confirm receipt of money or goods in exchange for sexual services within the past three months, and consciously define those activities as income-generating even if they did not consider sex work as their primary occupation [42]. At the initial study visit, participants’ HIV status was determined. Women who were found to be HIV-negative were eligible for the pilot study of HIV self-testing. Women who were found to be living with HIV were asked to complete a brief one-time questionnaire about HIV- and antiretroviral treatment (ART) history (following consent). Our overall target screening sample size was 200, with the expectation that approximately 80 women would be HIV-negative and eligible for the HIV self-testing pilot study.

### Recruitment

Participants were recruited by peer FSW outreach workers affiliated with the Nkaikela Youth Group, a non-governmental organization that provides services to FSW in and around Gaborone area. Women who received services from the Nkaikela Youth Group contacted the study team to self-refer for this pilot study, and referred other FSW to participate (after hearing about this study from the peer outreach workers following completion of our preceding qualitative study on HIV-self testing). Participants were then scheduled to come for a screening and entry visit.

### Study procedures

First, history and documentation of prior HIV testing (and test result) were obtained. Women who were not already documented to be HIV-positive were offered HIV counselling and testing using the OraQuick HIV self-testing kit, which has sensitivity of 90–98% and specificity of 98–100% [43, 44]. Trained study staff taught the participant how to use the oral HIV self-test (OraQuick), and the participant was observed as they performed the HIV self-test during the study visit; this initial test thus served as a training opportunity for participants.

#### HIV positive participants

Women who tested HIV-positive were counselled and referred for prompt confirmatory HIV testing and ART initiation if not already on treatment. Women with HIV also completed a brief one-time questionnaire about HIV and ART history and did not have subsequent study procedures.

#### HIV negative participants

Women whose entry visit oral HIV self-test was negative were offered two self-test kits to take home; one for them to test themselves, and a second kit to either share with another individual of their choosing or to use for another self-test on themselves. Women who declined taking the HIV self-testing kits home were to be invited for an in-depth interview to provide insights into reasons for declining. Women whose HIV self-test was negative were also counseled about and referred for tenofovir/emtricitabine oral pre-exposure prophylaxis (PrEP) to prevent HIV acquisition, which is available at no cost for key populations in Botswana.

#### Participants who accepted self-test kits

Participants who accepted the oral HIV self-test kits were provided with written step-by-step instructions from the manufacturer for using the HIV self-test, with diagrams and in participants’ preferred language (English or Setswana). Participants were also provided with a care card (one for each kit), on which the participant was asked to document the date and result of the HIV self-test. The care card also included guidance for next steps based on the self-test result, including contact information for rapid access to counselling and treatment services for participants who had a positive result (or to contact study staff if they had questions about how to perform the self-testing).

Women were counselled to test themselves using the HIV self-test kit approximately three months after the initial visit. They were given the option to decide whether or not study staff can send them a reminder text/SMS message to use the kit after three months. Women were counselled that they could use the second test kit to test themselves at any time that they wish—or, if they preferred, to test another adult who wished to and agreed to be HIV-tested, or to give the test kit to another individual with the written instructions for use. If the participant tested another individual, she was asked to give the individual a care card.

#### Participant follow up

Participants were asked to bring their care card to the follow-up appointment, in order to document whether or not they performed the self-test. Research staff emphasized to participants that they would not be judged negatively if they did not use the self-test kit, and highlighted that what was important was to understand whether the self-test was truly acceptable and feasible (to try to minimize social desirability bias in reporting of HIV self-testing). During the follow-up visit, participants were asked (using a structured questionnaire) about whether and when they performed the HIV self-test, the test result, their perceptions of self-testing, whether or not they were able to offer to test someone else with the extra self-test (and whom), and the advantages and the challenges/barriers associated with self-testing. Participants were reimbursed approximately $7 as compensation for transport and time at each visit, consistent with the Institutional Review Board approval.

### Outcomes and analysis

The primary outcome of interest was feasibility of HIV self-testing, defined as the proportion of women who used an HIV self-test during follow-up, among all women who were offered HIV self-test kits to take home. Secondary outcomes included the number of participants who passed on the extra self-test kit to someone else, and participant impressions of self-testing.

Statistical analysis was performed using STATA(version 16.0). FSW demographics and other characteristics at entry visit were summarized using frequencies, proportions for categorical variables; and means and ranges for continuous variables. Univariate analyses were conducted to evaluate the association between socio-demographic factors and reported use of HIV self-test kits.

## Results

Between June 2019 and September 2019, 200 FSW were recruited from Gaborone and surrounding areas. Of these 200 women, 199/200 (99.5%) had previously tested for HIV, 110 (55%) of whom had known HIV positive status at entry. Of the 90 women who underwent HIV self-testing at entry, an additional 5 tested positive and were referred for care and treatment. Hence, the overall prevalence of HIV was 58%. One hundred and seven (93%) of the 115 women living with HIV were taking ART (thus 107 [97%] of the 110 women who knew their positive status were taking ART).

**Fig 1:**
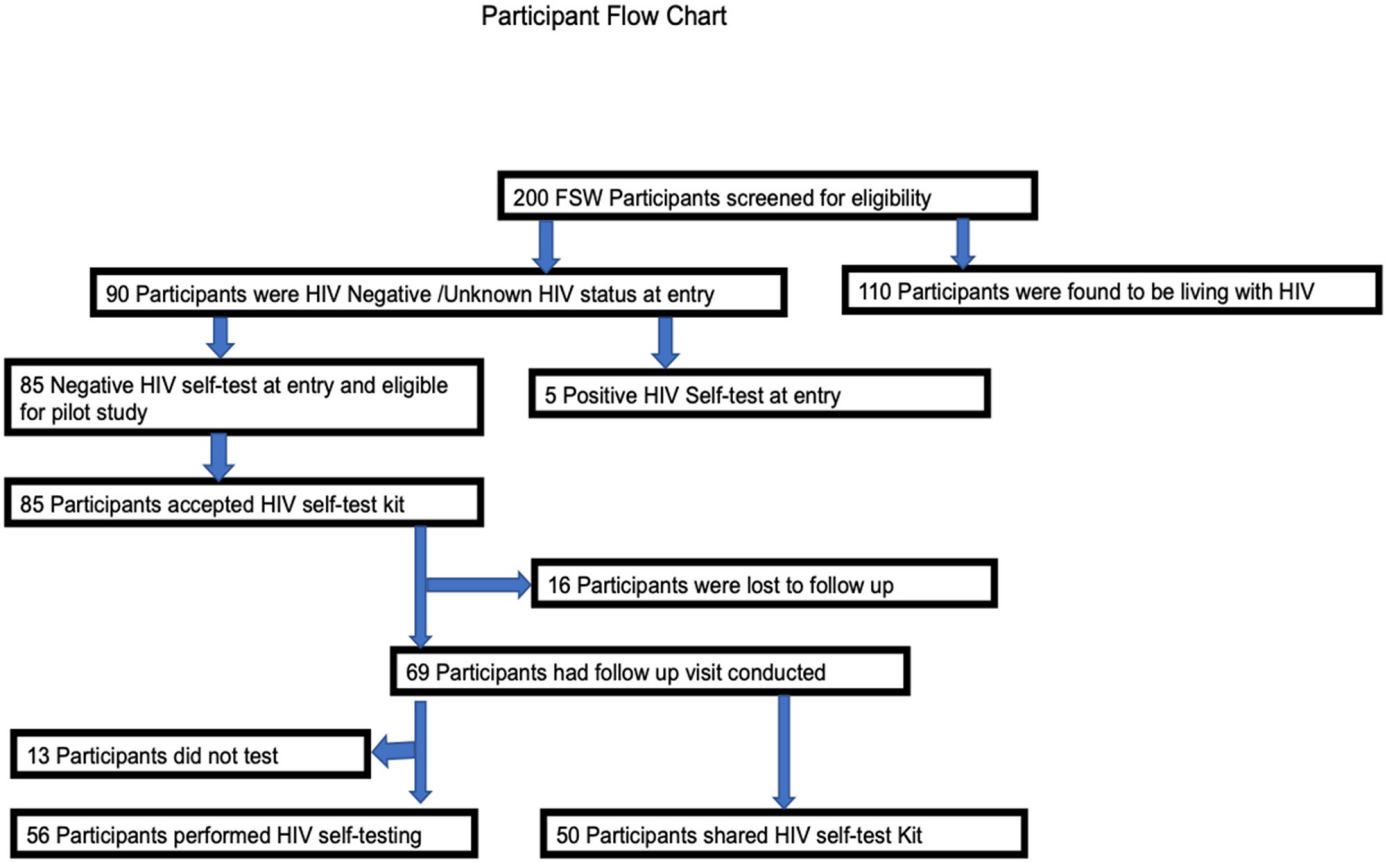
Participant flow chart.

Eighty-five (42%) of the 200 women recruited to the study were HIV-negative and were eligible to take part in the pilot study of HIV self-testing. Fig 1 shows the participant flow chart, and Table 1 the participant characteristics. None of the characteristics are statistically significant.

**Table 1:** Participants’ baseline characteristics.

Characteristic	All HIV-negative women (N = 85) n, %	All women with HIV (N = 115) n, %	All participants (N = 200) n, %	HIV Self-testing Odds ratio	95% CI
DEMOGRAPHICS					
Age					
Median age in years	29 (range 18–52)	37 (range 21–59)	34 (range 18–59)		
18–24	23 (27%)	6 (5%)	29 (15%)	2.4	0.3–17.0
25–34	45 (53%)	36 (31%)	81 (41%)	1.1	0.2–5.1
> 35	17 (20%)	73 (64%)	90 (46%)	REF	
Marital Status					
Married	2 (2%)	3 (3%)	5 (3%)	REF	
Single	61 (72%)	68 (59%)	129 (65%)	6	0.3–107.4
Co-habiting	20 (24%)	26 (23%)	46 (23%)	3	0.2–59.9
Divorced/separated	2 (2%)	11 (10%)	13 (7%)	1	0.02–50.4
Widowed	0	7(6%)	7 (4%)	N/A	N/A
Level of education					
None and Primary	10(12%)	31 (27%)	41 (21%)	REF	
Some secondary	44 (52%)	71 (62%)	115 (58%)	0.5	0.1–4.9
Completed secondary and Tertiary	31 (37%)	13 (11%)	44 (22%)	07	0.1–6.9
Occupation (in addition to sex work)					
Unemployed	55 (65%)	53 (46%)	108 (54%)	2.38	0.4–15.3
Self employed	8 (9%)	15 (13%)	23 (12%)	REF	
Formal employment	13 (15%)	18 (16%)	31 (16%)	2.25	0.2–22.1
Other	9 (11%)	29 (25%)	38 (19%)	2.50	0.2–38.6
Number of Children^a^					
One	13 (15%)	12 (10%)	25 (13%)	10	0.3–315.3
Two	42 (49%)	68 (59%)	110 (55%)	4.29	0.2–77.2
Three	25 (29%)	32 (28%)	57 (29%)	3.75	0.2–74.1
Four	5 (6%)	3 (3%)	8 (4%)	REF	
Participant Monthly Income^b^					
Less than US$100	19 (22%)	28 (24%)	47 (24%)	2.17	0.3–14.1
US$100-US$199	30 (35%)	35 (30%)	65 (33%)	1.67	0.4–7.9
US$200-US$299	16 (19%)	23 (20%)	39 (20%)	1.22	0.2–6.7
>US$300	20 (24%)	29 (25%)	49 (25%)	REF	
SEXUAL HISTORY					
Age at Sexual Debut (years)					
10–15	9 (11%)	32 (28%)	41 (21%)	REF	
16–19	63 (74%)	60 (52%)	123 (62%)	3.86	0.7–19.8
20–29	13 (15%)	23 (20%)	36 (18%)	1.2	0.2–8.8
Number of male sexual partners in last month					
0–10	69 (81%)	82 (71%)	151 (76%)	2.25	0.4–14.0
11–19	9 (11%)	8 (7%)	17 (9%)	3.5	0.2–51.9
> 20	7 (3%)	25 (21%)	32 (17%)	REF	
Drunk before most recent sex				0.81	0.2–2.7
Yes	33 (39%)	43 (37%)	76 (38%)		
No	52 (61%)	72 (63%)	124 (62%)		
Used condom during most recent sex				3.10	0.8–12.5
Yes	41 (48%)	85 (74%)	126 (63%)		
No	44 (52%)	30 (26%)	74 (37%)		
HIV-RELATED HISTORY					
Previously tested for HIV	85 (100%)	114 (99%)	199 (99%)	1	N/A
Previously diagnosed with HIV	N/A	110 (96%)	110(55%)	1	N/A
Any history of PrEP use	7 (8%)	0	7 (4%)	1.44	0.2–13.1
Currently taking PrEP	4/7 (57%)	N/A	4/7 (57%)	-	-
Currently on ART (among women who knew their diagnosis)	N/A	107/110 (97%)	N/A		

^a^All enrolled participants had children.

^b^Estimated US$ amount (US$1 = Pula10).

### Uptake of HIV self-testing

All 90 participants who were not already documented to be living with HIV accepted in-clinic HIV testing. They all performed assisted HIVST to the satisfaction of the study staff. All (100%) 85 women who tested HIV-negative accepted HIV self-testing kits to take home. Of these, 69/85 (81%) had follow up visits conducted whilst the remaining 16 women were lost to follow up despite multiple contact attempts. Fifty six (81%) of the 69 participants who followed up reported performing the HIV self-test, at a mean of 3.5 months after the initial visit (thus, 66% of all 85 women who were given HIV self-test kits followed up and reported using the test kit). All 56 participants who self-tested and followed up reported that the HIV self-test result was negative. None of the women used both test kits to test themselves. Of the 13 women who had a follow-up visit and who did not perform HIV self-testing, 6 either misplaced/lost the self-test kit, 3 were afraid to test, 3 changed their minds and no longer wanted to test, and one opted to test two people other than herself.

#### Testing experience

Of the 56 participants who had a follow up visit and self-tested; 20 tested alone; 19 tested in the presence of a family member, 12 tested in the presence of their non-client/primary partner, 3 tested in presence of a friend, and 2 tested with someone else. Forty nine participants HIV self-tested in their homes. None of the participants reached out to study staff for assistance with testing or interpreting the test result. Most participants 53/56 (95%) found performing oral HIV self-testing to be very easy whilst 3/56 (5%) felt it was fairly easy. None of the participants thought it was fairly difficult or difficult. Table 2 further explains the participants’ experiences of oral HIV self-testing.

**Table 2:** Participants’ experience on oral HIV self-testing.

Question on experience of oral HIV Self-testing	Yes	No	Maybe
Did you refer to the instructions provided when you last used the HIV self- test kit?	38/56 (69%)	18/56 (31%) (all remembered steps from the assisted testing at entry)	N/A
Did you call the study staff with questions about how to do the self-test?	0	56/56 (100%)	N/A
Did you call study staff about your test results?	55/56 (98%)	1/56 (2%)	N/A
Did you disclose your test results to anyone (other than our staff)?	46/56 (82%)	10/56 (18%)	N/A
Given a chance, would you use an HIV self-test kit in the future/ again?	64/69 (93%)	3/69 (4%)	2/69 (3%)
Would you recommend HIV self-testing to others?	58/69 (84%)	6/69 (9%)	5/69 (7%)

### Sharing of HIV self-test kits with others

Fifty (74%) of 69 participants shared at least one of the two oral HIV self-test kits with other individuals: 25 women shared the test kit with their non-client partners, 15 with a family member, and 8 with friends; 3 tested a client. One participant opted not to test herself and shared both her test kits, noting that she felt they needed them more than she did.

## Discussion

HIV testing is an important HIV prevention strategy, particularly for populations that are most vulnerable and hard to reach, We evaluated the feasibility of HIV self-testing among female sex workers in Gaborone Botswana. All HIV-negative women accepted self-test kits to take home. At least two thirds of women who were given oral HIV self-test kits to take home reported using them; uptake may have been higher, given that some of the ~20% of participants who were lost to follow-up after taking home HIVST kits may also have self-tested. Secondary distribution of the self-test kits by FSW to family members, friends and clients was also shown to be feasible and acceptable, with three quarters of women sharing a test kit with another individual. Our results suggest that HIV-self-testing by FSW (and dissemination of HIV self-test kits by FSW to others) is acceptable and feasible, and is a viable component of programs in sub-Saharan Africa that are scaling up HIV self-testing [18, 24, 45, 46].

Our participants were generally aware of their HIV risk, and over 70% had some secondary education. Despite this, only 4% of the participants had ever taken HIV PrEP, which was rolled out in Botswana starting in August 2018 for persons at increased risk of HIV infection, lower than uptake in other countries in the region [47, 48]. The reason for this low PrEP uptake may have been due to recent initiation of the PrEP program in key populations at the time. People taking PrEP due to their increased vulnerability to HIV are required to test for HIV frequently and could potentially also benefit from self-testing; HIV self-testing may also facilitate PrEP uptake, through additional awareness and engagement in care around HIV prevention.

Participants reported that oral HIV self-testing was very easy. No participants required any assistance with testing, consistent with findings from other research studies [28, 49]. Participants performed HIV self-testing mostly alone or in the presence of a trusted family member, consistent with existing literature, especially for the younger people [50]. Home was the most common place to test, also consistent with previous studies [18, 28, 51]. Linkage to care following HIV self-testing is an important consideration for persons who test positive [20]; we were not able to assess this linkage, as all women who tested and had follow up visit conducted reported having a negative test result.

Our findings suggest that secondary distribution of test kits by female sex workers is also feasible. Half of the participants who passed on a test kit did so to a non-client sexual partner, but distribution to clients was low, in contrast to other studies in the region [17, 31]. Reports have shown that sex workers in Botswana face high levels of violence and abuse [52], which could explain this finding. Distribution of HIV self-test kits to male partners is a promising HIV prevention strategy in the region as the men are known to infrequently engage in HIV Care [53–55].

We found high HIV prevalence (58%), particularly for a group of women who presented with interest in an HIV self-testing study. However, our sample was not random nor geographically dispersed, and HIV prevalence data should be interpreted with caution. ART coverage was very high (97% of women who knew their positive status and 93% of all women with HIV).

Limitations of our study included small sample size, sampling in one region of the country, and non-random sampling (although participant characteristics align well with those in other studies in the region [9, 17, 19, 22, 56]. Twenty percent of women given HIV self-test kits were lost to follow-up, possibly due to occurrence of the 2019 festive period (Christmas/New Year’s) just after the intended follow-up time. It is possible participants who were lost to follow up may not have used HIVST, and thus they may not have found HIVST to be as acceptable as the participants who remained in the study. We relied upon participant self-report of HIV self-test use and self-report of sharing of test-kits. The study did not collect any data from the other people who participants distributed self-test kits to; therefore their results and experiences of testing are unknown.

## Conclusion

The uptake and use of HIV self-testing by FSW in the Gaborone, Botswana region were high, and most women taking home HIV self-test kits also shared a test kit with a partner, family member or friend. HIV self-testing (and test kit distribution) by FSW appears to be a viable HIV testing approach in Botswana.

## Supporting information

S1 File HIV self testing entry form final version. (PDF)

S2 File HIV self testing exit form final version. (PDF)

S3 File Ikitse REDCap demographics CRF. (PDF)

S4 File Ikitse REDCap followup CRF. (PDF)

S5 File Ikitse data for sharing PlosOne rounded ages. (XLSX)
