# Supplementary material for: Feasibility of oral HIV self-testing in female sex workers in Gaborone, Botswana
Source: PLoS One. 2021 Nov 8;16(11):e0259508. doi: 10.1371/journal.pone.0259508 (PMC8575243; doi:10.1371/journal.pone.0259508)
Supplement: S1 File — (PDF) [file pone.0259508.s001.pdf]

Entry visit interview form

Instruction to study staff: Please tick the number of the correct answer or write the answer in the "answers" column			Answers
1	How old were you at your last birthday (in years)		
2	What is your marital status?	1.Single<br>2. Married<br>3. Cohabiting<br>4. Widowed<br>5. Divorced<br>6. Separated	
3	How many children do you have	1. 0<br>2. 1-2<br>3. 3-4<br>4. 5 or more	
4	What is your highest level of education	1. None<br>2. Primary School<br>3. Some Secondary education<br>4. Completed secondary education<br>5. Tertiary	
5	What is your country of origin	1. Botswana<br>2. Zimbabwe<br>3. Zambia<br>4. South Africa<br>5. Other	
6	What is your religion	1. None<br>2. Christianity<br>3. Muslim<br>4. Hindu<br>5. Traditional<br>6. Other	
7	What is your occupation	1. Formally employed<br>2. Unemployed<br>3. Self employed<br>4. Student<br>5. Other	
8	How much do you earn per month	1. P0-999<br>2. 1000-1999<br>3. 2000-2999<br>4. 3000 and more	
	Now I want to ask you some questions about your experiences seeking healthcare.		
9	When you need healthcare, where do you go to seek care? *(check all that apply)*	1. Community clinic<br>2. Government hospital<br>3. Faith-based hospital<br>4. Private doctor's office<br>5. Traditional healer	

		6. Other ________________<br>7. Prefer not to answer	
10	In the past 12 months, has there been a time when you needed healthcare but were unable to access it?	1. Yes<br>2. No<br>3. I don't know<br>4. Prefer not to answer	
11	What, if anything, has made it difficult for you to get the healthcare you need? *Do not read options, check all that apply*	1. I don't have transportation<br>2. I don't know where to get services<br>3. Proximity of clinic<br>4. I don't have money for services<br>5. Scheduled times that services are available are not good for my schedule<br>6. I don't have time<br>7. I don't feel comfortable going<br>8. I feel judged or discriminated against<br>9. I have not had difficulty getting the care I need<br>10. Other: _________<br>11. Prefer not to answer	
12	Please indicate whether you agree or disagree with the following statement:<br><br>5B04. I feel comfortable speaking with my medical care provider about working in sex work	1. Agree<br>2. Disagree<br>3. Prefer not to answer	
13	Please indicate whether you agree or disagree with the following statement:<br>I feel that my medical care providers judge me for working in sex work	4. Agree<br>5. Disagree<br>6. Prefer not to answer	
14a	Have you ever used pre-exposure prophylaxis (PrEP)?		
14b	If you used PrEP, are you still taking it? When did you start?		
15	Have you ever tested for HIV	1¤ Yes<br>2¤ No	
**Skip questions 16-18 if answer to 15 is "NO"**			
16	If yes to 10. when was your most recent HIV	Date_____________<br>MMM/YYYY	

	test	Don't know	
17	What was your most recent HIV test result	1. Negative<br>2. Positive<br>3. Indeterminate	
18	Where did you test for HIV, most recently?	1. Free standing VCT (e.g. Tebelopele)<br>2. Health clinic<br>3. Hospital<br>4. Other (specify)	
**Questions 19 to 29 are only for women who report already knowing that they are HIV-positive**			
19	When was the first time that you tested HIV-positive?	Date_______________<br>MMM/YYYY<br>Or: don't know	
20	Who have you told about your HIV status? (tick all that apply)	Partner(s)<br>Family member(s)<br>Friend(s)<br>Other (specify)<br>Nobody	
21	Have you ever had a CD4 count?	Yes<br>No<br>Don't know	
22a.	When was your last CD4 count	Date_______________<br>MMM/YYYY	
22b	What was the last CD4 count result	CD4 cell count______cells/mm^3<br>Or, don't know	
23	What was the source of data for the last CD4 count result	1. Review of participant medical record (e.g. IDCC card, OPD card)<br>2. Self-report<br>3. Both medical record and self-report	
24	Have you ever had your viral load checked?	Yes<br>No<br>Don't know	
25	When was your last viral load test	Date_______________<br>MMM/YYYY<br>Don't know	
26	What was your last viral load result	1. Undetectable<br>2. Detectable: ____ copies/mL<br>3. Don't know	
27	What was the source of data for the last viral load result	1. Review of participant medical record (e.g. IDCC card, OPD card)<br>2. Self-report<br>Both medical record and self-report	
28	Have you ever started antiretroviral treatment for your own health (not for PMTCT only)?	1. Yes<br>2. No	
29	Are you currently taking antiretroviral treatment	1. No<br>2. Yes	

	for your own health?		
**Following questions are for participants who report currently taking ART**			
30.	Which ART regimen are you taking	1. Dolutegravir based<br>2. Efavirenz based<br>3. Other (specify?)	
31	When did you first start ART for your own health	Date_______________<br>MMM/YYYY<br>Don't know	
32	What was the source of data for ART information	1. Review of participant medical record (e.g. IDCC card, OPD card, pill bottles)<br>2.Self-report<br>3. Both medical record and self-report	
**Sexual behavior questions:**<br>**I am going to ask you about your sexual relationships and about sexual practices. Please remember that all your answers are confidential**			
33	How old were you when you had sex for the first time?		
34	Over the past 12 months, how many times were you treated for asexually transmitted infection?		
35	Over the past 12 months, did you have an ongoing sexual relationship with more than one person at the same time? (including husband)		
36	Over the past 3 months, have you received money, transport, food/drink, or other goods in exchange for sex?		
37	In the past month, about how many different men have you had sex with?		
38	During the last (most recent) time you had sex, had you drunk alcohol before sex?		
39	During the last (most recent) time you had sex, did you or your partner use a condom?		
**Following questions are for participants with unknown HIV status/ HIV negative. Questions relate to HIV self testing**			
40	Was HIV pretest	1. Yes	

	counselling done	2. No	
41	Comment on issues identified during HIV pretest counselling		
**Study staff to observe participant performing HIV self-testing after training her**			
42	What was the HIV test result	1. Positive<br>2. Negative<br>3. Indeterminate	
**If positive refer for confirmatory HIV testing at local clinic and ART initiation, if negative refer for PrEP.**<br>**Provide post HIV counselling. A negative result is negative and a positive result requires confirmation.**			
43	Based on the study staff observation, how well was the participant able to perform the HIV self test	1. Very well<br>2. Fairly well<br>3. With some difficulty<br>4. Not able to do the test	
44	Was participant referred for further HIV testing	1. Yes<br>2. No	
45	To what extent do you understand how HIV self testing is done	1. Very well<br>2. Fairly well<br>**3.** Slightly understand<br>4. Not at all	
46	To what extent do you require further training?	1. I do not require any more training<br>2. I would require some more training<br>3. I would require a lot more training	
47	If you think you need more training, what type of training do you need?		
48	Did participant accept the HIV self test kits to take home?	1. Yes<br>2. No	
	**If yes, provide participant with a care card for each self test kit. Explain that she needs to self test in about 4 months and also test someone else if you wish, and with their permission (and give them the care card). Provide participant with approximate date of testing and ensure that she understands how to contact staff with questions, including if the test is positive**		
	**If No to Q 48, administer questionnaire for participants who decline self test kit to take home.**		
