# Supplementary material for: Feasibility of oral HIV self-testing in female sex workers in Gaborone, Botswana
Source: PLoS One. 2021 Nov 8;16(11):e0259508. doi: 10.1371/journal.pone.0259508 (PMC8575243; doi:10.1371/journal.pone.0259508)
Supplement: S2 File — (PDF) [file pone.0259508.s002.pdf]

Follow up visit/Exit Interview form

**Instruction to study staff: Please tick the number of the correct answer or write the answer in the "answers" column**			**Answers**
1	Did you do the HIV self test	1. Yes<br>2. No	
2a	Have you done any other HIV test since your last study visit	1. Yes<br>2. No	
2b	If Yes specify	________	
**Question 3- 5 are for participants who did not do the HIV self-test**			
3	If you did not do the HIV self test, Please explain why? Choose all that apply	1. I did not understand how to do it<br>2. I was afraid to test<br>3. I changed my mind about testing (explain what made you change your mind)<br>4. I forgot<br>5. Other Specify	
4	What is the **main** reason that you did not self-test?	1. I did not understand how to do it<br>2. I was afraid to test<br>3. I changed my mind about testing (explain what made you change your mind)<br>4. I forgot<br>Other Specify	
5	What do you think could have improved your chances of HIV self testing		
**Questions 6-14 for participants who HIV self-tested**			
6	If yes, when did you do the test?	Date_______________<br>DD/MMM/YYYY	
7	If yes what was the test result	1. Positive<br>2. Negative<br>3. Indeterminate	
8	Where did you do the HIV self test	1. At home (where I currently live)<br>2. At my workplace (describe workplace type)<br>3. At a friend's house<br>4. At my family's house<br>5. Other (describe)	
9	How easy or hard was it to read the test result/do the self-test?	1. Very Easy<br>2. Fairly easy<br>3. Somewhat difficult<br>4. Very difficult	
10	If responded anything other than "very easy": What were the challenges?	1. Did not know how to collect the sample<br>2. Did not understand	

	Choose all that apply	how to read the result<br>3. Other Specify	
11	Did you refer to the instructions provided when you last used the HIV self-test kit?	1. Yes<br>2. No<br>3. Prefer not to answer	
12	If no to above; Why did you not use the provided instructions?	4. Too complex, didn't understand<br>5. Cannot read<br>6. Remembered steps from study staff training<br>7. Called the study staff<br>8. Other: ____________<br>9. Prefer not to answer	
13	Who was present when you tested	1. No-one<br>2. Partner<br>3. Friend<br>4. Family member (other than partner)<br>5. Other (specify)	
14a	Did you disclose your test results to anyone (other than our staff)?	1. Yes<br>2. No	
14b	If yes specify		
15a	Did you test anyone else for HIV?	1. Yes<br>2. No	
15 b	If yes to above, who did you test?	1. Client (sex partner)<br>2. Partner (not client)<br>3. Family member<br>4. Work mate<br>5. Friend<br>6. Other Specify	
15 c	If No, why not?		
16	Did you call the study staff with questions about how to do the self-test?	1. Yes<br>2. No	
17	Did you call the study staff about the HIV self-test result?	1. Yes<br>2. No	
18	Did the participant bring back the HIV self-test kit	1. Yes<br>2. No	
19	Was the test kit opened/used	1. Yes<br>2. No	
**Questions 20-25 are on future directions**			
20a	Would you recommend	1. Yes	

	HIV self testing to others	2. No<br>3. Maybe	
20b	Why?		
21	How do you think eligibility for self testing should be determined	1. Prescribed by health providers<br>2. Provided (with training) by trained peers/lay personnel<br>3. Open access/ unsupervised, with anyone wishing to self test accessing the test kits without health personnel assistance.<br>4. Other Specify	
22	How do you suggest HIV-self test kits should be distributed	1. At clinics<br>2. Outreach<br>3. Over the counter at pharmacies or shops<br>4. Other Specify	
23	How do you suggest HIV self testing be done in future	1. Person self tests in private<br>2. Supervised HIV self testing<br>3. Other Specify	
24	What are the potential challenges of using HIV self test kits		
25	Given a chance, would you use an HIV self test kit in the future/ again	1. Yes<br>2. No<br>Maybe	
26	What was your experience of testing someone else for HIV- useful, problematic etc?		
