# Supplementary material for: Feasibility of oral HIV self-testing in female sex workers in Gaborone, Botswana
Source: PLoS One. 2021 Nov 8;16(11):e0259508. doi: 10.1371/journal.pone.0259508 (PMC8575243; doi:10.1371/journal.pone.0259508)
Supplement: S3 File — (PDF) [file pone.0259508.s003.pdf]

cord_id	Record_Id	text		
	2	subject_id	Subject_ID	text, Required, Identifier
	3	age	1.How old were you at your last birthday (in years)	text, Required
	4	marital_status	2.What is your marital status?	radio, Required<br>1 Single<br>2 Married<br>3 Cohabiting<br>4 Widowed<br>5 Divorced<br>6 Separated<br>7 Do not want Answer
	5	number_children_you_have	3.How many children do you have	radio, Required<br>1 0<br>2 1-2<br>3 3-4<br>4 5 or more<br>5 Do not want to answer
	6	level_education	4.What is your highest level of education	radio, Required<br>1 None<br>2 Primary School<br>3 Some Secondary education<br>4 Completed secondary education<br>5 Tertiary<br>6 Do not want to answer
	7	origin_country	5.What is your country of origin	radio, Required<br>1 Botswana<br>2 Zimbabwe<br>3 Zambia<br>4 South Africa<br>5 Other<br>6 Do not want to answer
