# Supplementary material for: Feasibility of oral HIV self-testing in female sex workers in Gaborone, Botswana
Source: PLoS One. 2021 Nov 8;16(11):e0259508. doi: 10.1371/journal.pone.0259508 (PMC8575243; doi:10.1371/journal.pone.0259508)
Supplement: S4 File — (PDF) [file pone.0259508.s004.pdf]

do_hiv_self_tet1.Did you do the HIV self testyesno

1	Yes
0	No

82date_self_test_done

Show the field ONLY if:
[do_hiv_self_tet] = '1'
1a.Date self test donetext

83date_estimated

Show the field ONLY if:
[do_hiv_self_tet] = '1'
1b.Is date estimatedradio

1	No
2	Yes, estimated day
3	Yes, estimated month and day
4	Yes estimated year, month and day

84result_hiv_test

Show the field ONLY if:
[do_hiv_self_tet] = '1'
1c.What are the results of this HIV self testradio

1	Postive
2	Negative
3	I don't remember
4	I don't want to answer

85explain_why_hiv_test

Show the field ONLY if:
[do_hiv_self_tet] = '0'
2.If you did not do the HIV self test, Please explain why? Choose all that applycheckbox

1	explain_why_hiv_test___1	I did not understand how to do it
2	explain_why_hiv_test___2	I was afraid to test
3	explain_why_hiv_test___3	I changed my mind about testing (explain what made you change your mind)
4	explain_why_hiv_test___4	I forgot
5	explain_why_hiv_test___5	Other Specify

86other_explain

Show the field ONLY if:
[explain_why_hiv_test(3)] = '1' or [explain_why_hiv_test(5)] = '1'
2a.Othertext

87main_reason_not_self_test

Show the field ONLY if:
[do_hiv_self_tet] = '0'
3.What is the main reason that you did not self-test?radio

1	I did not understand how to do it
2	I was afraid to test

3	I changed my mind about testing (explain what made you change your mind)
4	I forgot
5	Other Specify

88other_reason_not_selftest

Show the field ONLY if:
[main_reason_not_self_test] = '5' or [main_reason_not_self_test] = '3'
3a.Othertext

89chances_self_testing

Show the field ONLY if:
[do_hiv_self_tet] = '0'
4.What do you think could have improved your chances of HIV self testingnotes

90hiv_test_done5.Have you done any other HIV test since your last study visityesno

1	Yes
0	No

91date_self_test

Show the field ONLY if:
[hiv_test_done] = '1'
6.If yes, when did you do the test?text (date_dmy)

92self_test_result

Show the field ONLY if:
[hiv_test_done] = '1'
7.If yes what was the test resultradio

1	Positive
2	Negative
3	Indeterminate

93place_hiv_self_test

Show the field ONLY if:
[do_hiv_self_tet] = '1'
8.Where did you do the HIV self testradio

1	At home (where I currently live)
2	At my workplace (describe workplace type)
3	At a friend's house
4	At my family's house
5	Other (describe)

94other_places

Show the field ONLY if:
[place_hiv_self_test] = '5' or [place_hiv_self_test] = '2'
8a.Othertext

95difficulties_read_test

Show the field ONLY if:
[do_hiv_self_tet] = '1' and [do_hiv_self_tet] = '1'
9.How easy or hard was it to read the test result/do the self-test?radio

1	Very Easy
2	Fairly easy

3	Somewhat difficult
4	Very difficult

96challenges

Show the field ONLY if:
[do_hiv_self_tet] = '1' and [do_hiv_self_tet] = '1'
10.If responded anything other than "very easy": What were the challenges?Choose all that applycheckbox

1	challenges___1	Did not know how to collect the sample
2	challenges___2	Did not understand how to read the result
3	challenges___3	Other Specify

97other_challenges

Show the field ONLY if:
[challenges(3)] = '1'
10a.Othertext

98refer_instructions

Show the field ONLY if:
[do_hiv_self_tet] = '1' and [do_hiv_self_tet] = '1'
11.Did you refer to the instructions provided when you last used the HIV self- test kit?radio

1	Yes
2	No
3	Prefer not to answer

99not_use_instructions

Show the field ONLY if:
[do_hiv_self_tet] = '1'
12.If no to above; Why did you not use the provided instructions?radio

1	Too complex, didn't understand
2	Cannot read
3	Remembered steps from study staff training
4	Called the study staff
5	Other
6	Prefer not answer

100other_not_use_instructions

Show the field ONLY if:
[not_use_instructions] = '5'
12a.Othertext

101present_when_tested

Show the field ONLY if:
[do_hiv_self_tet] = '1'
13.Who was present when you testedradio

1	No-one
2	Partner
3	Friend
4	Family member (other than partner)
5	Other (specify)

102other_present_tested

Show the field ONLY if:
[present_when_tested] = '5'
13a.Othertext

103disclose_test_results

Show the field ONLY if:
[do_hiv_self_tet] = '1'
14aDid you disclose your test results to anyone (other than our staff)?yesno

1	Yes
0	No

104disclose_specify

Show the field ONLY if:
[disclose_test_results] = '1'
14b.If yes specifytext

105test_anyone_else

Show the field ONLY if:
[do_hiv_self_tet] = '1' or [hiv_test_done] = '0'
15a.Did you test anyone else for HIV?yesno

1	Yes
0	No

106who_did_you_test

Show the field ONLY if:
[test_anyone_else] = '1'
15b.If yes to above, who did you test?checkbox

1	who_did_you_test___1	Client (sex partner)
2	who_did_you_test___2	Partner (not client)
3	who_did_you_test___3	Family member
4	who_did_you_test___4	Work mate
5	who_did_you_test___5	Friend
6	who_did_you_test___6	Other Specify

107other_who_you_tested

Show the field ONLY if:
[who_did_you_test(6)] = '1'
15c.Othertext

108why_not

Show the field ONLY if:
[test_anyone_else] = '0'
15d.If No, why not?text

109call_study_staff

Show the field ONLY if:
[do_hiv_self_tet] = '1'
16.Did you call the study staff with questions about how to do the self-test?yesno

1	Yes
0	No

110call_about_result

Show the field ONLY if:
[do_hiv_self_tet] = '1'
17Did you call the study staff about the HIV self-test result?yesno

1	Yes
0	No

111bring_back_kit

Show the field ONLY if:
[do_hiv_self_tet] = '1'
18.Did the participant bring back the HIV self-test kityesno

1	Yes
0	No

112why_not_bring_back

Show the field ONLY if:
[bring_back_kit] = '0'
18a.If no why nottext

113kit_opened_used

Show the field ONLY if:
[bring_back_kit] = '1'
19.Was the test kit opened/usedyesno

1	Yes
0	No

114recommend_self_testing20a.Would you recommend HIV self testing to othersradio

1	No
2	Yes
3	Maybe

115why

Show the field ONLY if:
[recommend_self_testing] = '1' or [recommend_self_testing] = '2' or [recommend_self_testing] = '3'
20b.Whytext

116determining_eligibility21.How do you think eligibility for self testing should be determinedcheckbox

1	determining_eligibility___1	Prescribed by health providers
2	determining_eligibility___2	Provided (with training) by trained peers/lay personnel
3	determining_eligibility___3	Open access/ unsupervised, with anyone wishing to self test accessing the test kits without health personnel assistance.
4	determining_eligibility___4	Other Specify

117other_determining_eligibility

Show the field ONLY if:
[determining_eligibility] = '4'
21a.Othertext

118distribution_kits22.How do you suggest HIV- self test kits should be distributedcheckbox

1	distribution_kits___1	At clinics
2	distribution_kits___2	Outreach
3	distribution_kits___3	Over the counter at pharmacies or shops
4	distribution_kits___4	Other Specify

119other_distribution_kits

Show the field ONLY if:
[distribution_kits] = '4'
22a.Othertext

120future_suggestion23.How do you suggest HIV self testing be done in futureradio

1	Person self tests in private
2	Supervised HIV self testing
3	Other Specify

121other_future_suggestion

Show the field ONLY if:
[future_suggestion] = '3'
23a.Othertext

122potential_challenges24.What are the potential challenges of using HIV self test kitsnotes

123use_hiv_self_test25.Given a chance, would you use an HIV self test kit in the future/ againradio

1	Yes
2	No
3	Maybe

124why_use_self_test_again25a.Whytext

125experience_testing_someone26.What was your experience of testing someone else for HIV- useful, problematic etc?text

126pretest_counselling_done27.Was HIV pretest counseling done todayyesno

1	Yes
0	No

127comment_counselling28. Comment on issues identified during pretest counseling?text

128unigold_result
Section Header: *Unigold results*

Resultradio (Matrix)

1	POS
2	NEG

129determine_results
Section Header: *Determine Results*

Resultsradio (Matrix)

1	Positive
2	Negative

130rapid_test
Section Header: *Double rapid test*

Double rapid testradio (Matrix)

1	Positive
2	Negative
3	Indeterminate

131confirmatory_test

Show the field ONLY if:
[rapid_test] = '3'
31.Was participant referred for confirmatory HIV testing?yesno

1	Yes
0	No

132date_confirmatory_test

Show the field ONLY if:
[rapid_test] = '3' and [confirmatory_test] = '1'
31a.Date of confirmatory HIV testing?text

133date_estimated_confirmatory

Show the field ONLY if:
[rapid_test] = '3' and [confirmatory_test] = '1'
31b.Is date estimatedradio

1	No
2	Yes, estimated day
3	Yes, estimated month and day
4	Yes estimated year, month and day

134why_comfirmatory_not_done

Show the field ONLY if:
[confirmatory_test] = '0'
32.If No , why nottext

135confirmatory_hiv_done

Show the field ONLY if:
[rapid_test] = '3' and [confirmatory_test] = '1'
33.If YES, which tests doneradio

1	Elisa
2	Western Blot
3	DNA PCR

136final_hiv_result

Show the field ONLY if:
[rapid_test] = '3' and [confirmatory_test] = '1'
34.What is the final HIV test resultradio

1	Positive
2	Negative

137date_final_results

Show the field ONLY if:
[rapid_test] = '3' and [confirmatory_test] = '1'
34a.Date for final HIV resulttext

138follow_up_complete
Section Header: *Form Status*

Complete?dropdown

0	Incomplete
1	Unverified
2	Complete
